# Impact of COVID-19 pandemic, and the mediating role of hospital caseload and severity on mortality of hospitalised tuberculosis patients in Thailand

**DOI:** 10.1186/s41256-025-00437-7

**Published:** 2025-08-25

**Authors:** Nyi Nyi Zayar, Rassamee Chotipanvithayakul, Alan Frederick Geater, Kyaw Ko Ko Htet, Chumpol Ngamphiw, Virasakdi Chongsuvivatwong

**Affiliations:** 1 Department of Epidemiology, Faculty of Medicine, Prince of Songkla University, 15 Kanjanavanich Road, Hat Yai, Songkhla, 90110 Thailand; 2 National Biobank of Thailand, National Science and Technology Development Agency, Khlong Luang, Pathum Thani 12120 Thailand

**Keywords:** Tuberculosis, COVID-19, Hospital caseload, Mortality, Severity, Charlson comorbidity index

## Abstract

**Background:**

The COVID-19 hospital caseload indicates the quality of hospital care, as resources were redirected to address the surge in COVID-19 cases. The study aimed to evaluate the impact of COVID-19 hospital caseload on hospital tuberculosis (TB) case fatality rate (CFR) mediated by the TB caseload and severity of patients.

**Methods:**

A retrospective analysis of TB patients’ hospital admission data in Thailand extracted from the Thai Health Information Portal database between January 2017 and September 2022. Charlson Comorbidity Index (CCI) was used to determine the severity of hospitalised TB patients. An interrupted time series analysis, lag time analysis and serial mediation analysis were done.

**Results:**

During COVID-19 pandemic, there was a 12.9% decrease in monthly hospital TB caseload, and a 14.1% increase in monthly TB hospital CFR compared to the counterfactual scenario had there been no COVID-19. COVID-19 hospital caseload had a strong negative correlation with TB hospital caseload (r = − 0.60, *p*-value = < 0.001), but a strong positive correlation with TB hospital CFR (r = 0.74, *p*-value = < 0.001) during the same month. An increase in average CCI score of 0.1 was associated with an increase of 2.3 deaths per 100 TB admissions. After adjusting the TB caseload and CCI of TB patients admitted to the hospital, no association was found between COVID-19 hospital caseload and the hospital CFR of TB patients.

**Conclusions:**

The increase in TB hospital CFR during COVID-19 pandemic was likely driven by a higher proportion of severe cases being admitted, rather than a decline in hospitals’ quality of care.

**Supplementary Information:**

The online version contains supplementary material available at 10.1186/s41256-025-00437-7.

## Background

Globally, tuberculosis (TB) is the leading cause of mortality, with more than 1.5 million deaths caused by a single pathogen in 2022 [1]. Recently, COVID-19 has infected 700 million people and led to 7 million deaths globally [2]. The overwhelming workload of COVID-19 treatment and control measures disrupted the healthcare systems, including the TB healthcare services. The most obvious impact was an 18% reduction in the newly diagnosed TB reported in 2020 compared to 2019 [1]. Furthermore, the estimated number of TB-related deaths increased from 1.4 million in 2019 to 1.6 million in 2021 [1]. Thailand is one of the top 30 countries with the highest incidence rate of TB burden in the world [1]. Between 2020 and 2024, the country recorded over 4.7 million COVID-19 infections and nearly 35,000 deaths related to COVID-19 [2]. The impact of COVID-19 affected the Thailand TB control program as well. Global TB Report in 2022 stated that Thailand is one of the top ten countries that decreased TB case notifications, and undiagnosed and untreated TB in 2021, compared to 2019 [1].

Investigating how COVID-19 impacted the use of healthcare services and treatment outcomes of TB patients is crucial for preparing TB healthcare services for future pandemics. Studies in Turkey [3] and China [4] have already explored the effect of COVID-19 on TB healthcare service using National TB Program data from their own country, primarily from outpatient departments. The studies showed a decrease in TB incidence but increased mortality due to the disruption of healthcare service resources, including TB diagnosis and treatment programs. Nonetheless, these studies did not primarily focus on the impact of the pandemic on healthcare services to TB patients admitted to hospitals. A study showed that the impact of COVID-19 pandemic on hospital healthcare facilities was greater than that on primary healthcare facilities [5]. Nearly 45% of sputum smear-positive patients required hospitalisation for treatment in Thailand [6]. Despite the high hospitalisation rate, there is limited information about the impact of COVID-19 pandemic on morbidity and mortality of TB patients admitted to hospitals. Understanding this aspect will help identify weaknesses and develop strategies to maintain effective TB care in hospitals during global health crises.

Previous studies reported that the caseload of hospitalised patients with non-COVID-19 diseases was decreased [4], while hospital mortality was increased during COVID-19 pandemic [7–9]. These may be explained by two research hypotheses. First, the high caseload of COVID-19 patients shifted the healthcare resources to COVID-19 department, resulting in reduced hospital services for other diseases and increased mortality [10]. The other is that non-severe patients who did not require special treatment avoided going to the hospital due to fear of contracting COVID-19 infection, leaving only severe patients who required special care to present in the hospitals, and subsequently leading to higher mortality rates [7–9]. So far, none of the studies could disentangle these complex causal chains.

Our study intended to test these hypotheses based on two explanatory factors: the caseload of COVID-19 patients presenting in the hospital, and the hospital admission of TB patients with severe conditions. The hospital caseload of COVID-19 patients can serve as an indicator of the quality of healthcare services in hospitals, as resources were redirected to address the surge in COVID-19 cases [11, 12]. Understanding the effect of COVID-19 hospital caseload on TB case fatality rate (CFR) in hospital and the mediating effect of TB caseload and severity of TB patients admitted to hospital between these two factors would inform healthcare planners to relocate resources, mitigate the excessive workload and alleviate unfavorable outcomes of TB patients in future pandemics in Thailand. The Thai Health Information Portal (THIP) is a database of hospitalised patients set up in 2017 [13]. It contains hospital admission data of more than 34 million patients admitted to hospitals in Thailand. This system allows us to test the above two hypotheses since the information based on the THIP database, caseload of COVID-19 hospitalised TB and severity of the TB cases could be computed. Our objectives were (1) to evaluate the impact of COVID-19 pandemic on hospital caseload and CFR of TB patients admitted to the hospital, and (2) to explore the effect of COVID-19 hospital caseload on in-hospital TB CFR mediated by the TB caseload and the severity of TB patients.

## Methods

### Study design and population

The retrospective study analysed TB hospitalisation data in the THIP database [13]. It recorded hospital admission data in Thailand from January 2017 to September 2022.

### Study setting and population

Thailand is located in the Southeast Asia Region with a total population of 716,983,332. It comprises 331 superdistrict areas, which are delineated by larger administrative boundaries rather than natural features, under the jurisdiction of 13 health regions throughout the country (Supplementary Fig. 1). This study included TB patients newly admitted to hospitals in 331 superdistrict areas in Thailand between January 2017 and September 2022.

### Situation of COVID-19 pandemic in Thailand

In Thailand, the Royal Thai Government announced a Decree on Public Administration in an Emergency Situation on 25th March 2020 [14]. Following that, the Royal Thai Government Gazette published an announcement on a full-scale national lockdown between April and June 2020 [15]. Thailand has experienced five outbreaks of COVID-19 including first wave (March–May 2020), second wave (December 2020 – March 2021), third wave dominant by Alpha variant (April – June 2021), fourth wave dominant by Delta variant (June—December 2021), and fifth wave dominant by Omicron variant (January – September 2022) as shown in Supplementary Fig. 2 [16]. Nationwide lockdown was implemented during the first outbreak by closing department stores, restaurants, markets and schools, restricting attendance to marriage and funerals, and travel overseas. In the following outbreaks, only a partial lockdown in affected areas was applied due to the economic impact of a nationwide lockdown [17].

### Data sources

TB and COVID-19 hospital admission data were extracted from the THIP Database, which is a data warehouse cooperation between National Health Security Office (NHSO), Prince of Songkla University, National Science and Technology Development Agency (NSTDA) and National Biobank of Thailand (NBT) [13]. This cooperative project retrieved and deidentified patients’ hospital admission data submitted to NHSO to be opened for researchers in Thailand to analyse and inform policy to improve the health system. THIP Database comprises hospital admission data of patients admitted to 13,136 health facilities across the country between January 2017 and September 2022. The THIP database stores daily records of individual patient’s information admitted to the hospital with a primary diagnosis of TB or COVID-19. In this study, we used the background data of the patients, including age and sex, individual record of hospital admission, date of admission, date of discharge, discharge status, and comorbid diseases identified by the International Classification of Diseases, tenth revision (ICD-10) code [18].

**Fig. 1:**
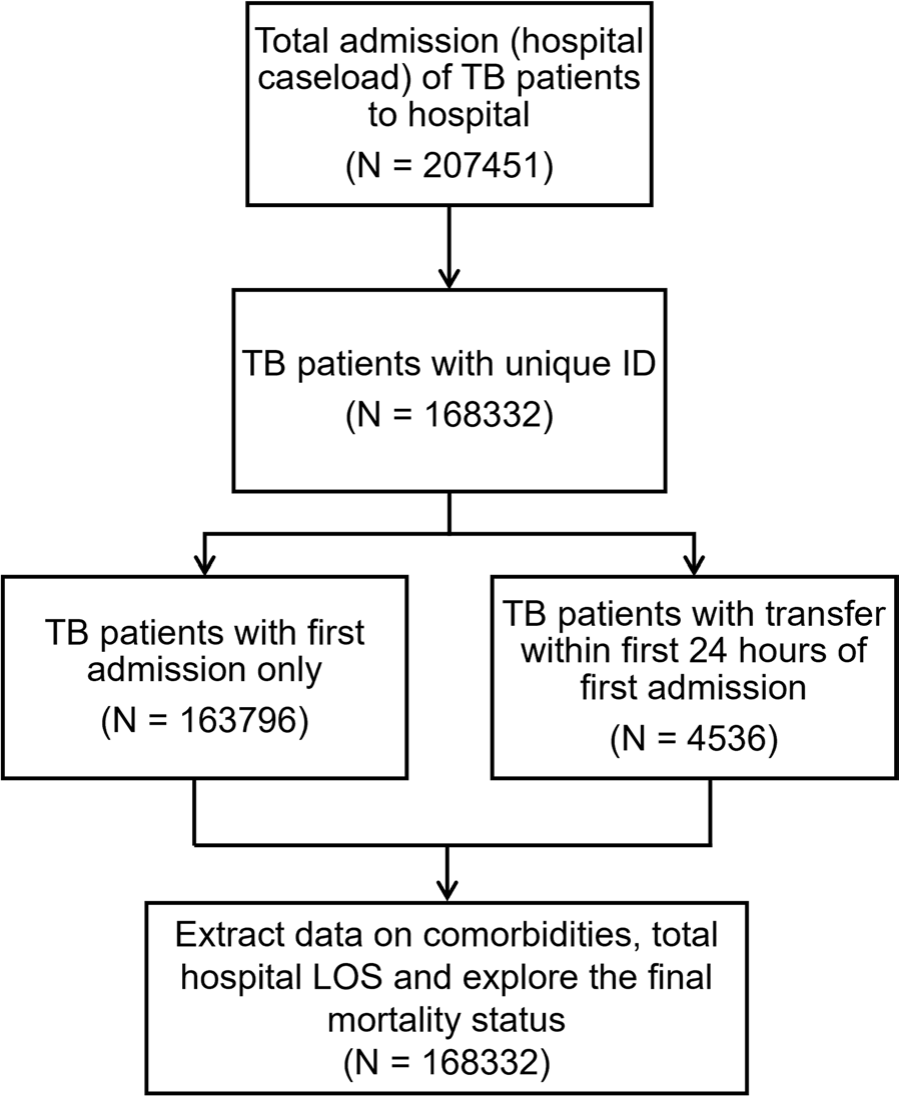
Flow diagram of selection of TB patients newly admitted to hospitals. TB, Tuberculosis

Figure 1 shows the flow diagram of the selection process of TB patients newly admitted to hospitals in Thailand. A total of 207,451 hospital admissions of 168,332 TB patients were identified, corresponding to 168,332 unique TB patients. Among them, 163,796 patients were admitted for a single time only, while 4,536 were transferred to another health facility within the first 24 h of their initial admission. Data on associated comorbidities, hospital LOS, and hospital discharge status during initial admission and transfer were compiled to determine the final hospital outcomes.

### Variables

#### Independent variables

The background characteristics of TB patients included age and sex. Age was categorised into under 15 years old, 15 to 59 years old and 60 years and above. Sex was categorised into male and female. Different types of TB identified were based on ICD-10 codes from A15 to A19. The severity of TB patients was defined using the Charlson Comorbidity Index (CCI), which is a method for predicting mortality by weighting comorbid conditions to measure the burden of disease [19]. COVID-19 caseload is the monthly number of COVID-19 patients admitted to hospitals from 1st March 2020 to 30th September 2022.

#### Outcome variables

TB hospital caseload is defined as the monthly number of TB patients admitted to the hospitals from 1st January 2017 to 30th September 2022. The CFR of TB patients is defined as the monthly number of TB patients who died in the hospital per 100 admissions of TB patients [20].

### Data analysis

Data analysis was performed using R software version 4.1.3 [21]. The background information of patients admitted to the hospital with a primary diagnosis of TB was shown as frequency and percentage for categorical variables, and mean with standard deviation for continuous variables. The comparison of background characteristics between new TB patients admitted to the hospital before and during COVID-19 pandemic was done using the chi-square test and student “*t*” test.

#### Interrupted time series analysis

Interrupted time series (ITS) analysis was performed to determine the change in monthly TB caseload and CFR during the overall period of COVID-19 pandemic. The impact of COVID-19 pandemic on outcomes of interest was determined by the percent difference between the observed value of the outcome during the pandemic and the predicted value of the outcome had there been no COVID-19 pandemic. The counterfactual values were predicted using the Poisson regression model or negative binomial regression model, depending on whether there was an overdispersion problem, using the following Eq. (1) [22].

1 $$Y_{t} = \beta_{0} + \beta_{1} T + \beta_{2} D_{c} + \beta_{3} T_{c} + offset\left( {\log \left( P \right)} \right) + e_{t}$$

where $Y_{t}$ represents the outcome which is the average monthly TB hospital caseload (or) average monthly number of TB deaths in hospital; $T$ is a continuous variable indicating the time in months passed from the start of the observation period (1st January 2017); $D_{c}$ is a binary variable indicating the period before the announcement on COVID-19 emergency state (0) or during the COVID-19 pandemic after national announcement (1); $T_{c}$ is a continuous variable counting the number of months started from the first outbreak of COVID-19 period (1st March 2020) at time $t$. The term $e_{t}$ represents the random error. $P$ represents the number of TB patients admitted to the hospital during the same month as the number of TB deaths in the hospital to estimate the monthly TB in-hospital CFR. The autocorrelation function (ACF) and partial autocorrelation function (PACF) were used to check for autocorrelation of the error terms in the models [23]. No significant autocorrelation was found in models for both TB hospital caseload (Supplementary Fig. 3) and TB CFR (Supplementary Fig. 4). Additional analysis on the impact of COVID-19 caseload on TB in-hospital caseload and CFR stratified by 13 health regions of Thailand were done (Supplementary Table. 2).

The lag time association between monthly COVID-19 caseload with monthly TB caseload and monthly TB CFR was identified using the cross-correlation function at the national level (Supplementary Fig. 5 and 7) and also separately for different health regions (Supplementary Fig. 6 and 8).

The graphical presentation of the predicted time series against the observed time series was plotted using R. Counterfactual lines during the COVID-19 pandemic were predicted to approximate the value of the outcome had there not been a COVID-19 pandemic. The percent difference in the average number of monthly TB caseload and CFR between observed and counterfactual data was used to estimate the impact of COVID-19, computed by the following formula (2) [22].

2 $$\frac{{{\text{Observed}}\,{\text{value}}\,{\text{with}}\,{\text{COVID }}\,19{ }\,{\text{pandemic }}{-}\,{\text{Counterfactual}}\,{\text{value}}\,{\text{without}}\,{\text{COVID }}19\,{\text{pandemic}}}}{{{\text{Counterfactual}}\,{\text{value }}\,{\text{without }}\,{\text{COVID }}19\,{\text{pandemic}}}} \times 100\%$$

#### Serial mediation analysis of TB caseload and severity of TB patients between COVID-19 hospital caseload and in-hospital TB CFR

We used the serial mediation model [24] as shown in Fig. 4 to explore the serial and individual mediation effects of TB caseload and CCI score between COVID-19 hospital caseload on in-hospital TB CFR across the country from March 2020 until September 2022. In the model, the continuous data, including severity of TB patients, in-hospital TB CFR and their associated trends were aggregated into monthly averages, and COVID-19 and TB hospital caseloads were aggregated into monthly totals.

In the model, three mediation tests were performed simultaneously. M_1_ indicates the TB caseload dependent mediation pathway between COVID-19 hospital caseload and TB CFR (COVID-19 hospital caseload → TB caseload → in-hospital TB CFR). M_2_ shows both TB caseload and CCI dependent serial mediation pathway between COVID-19 hospital caseload and TB CFR (COVID-19 hospital caseload → TB caseload → CCI → in-hospital TB CFR). And M_3_ expresses the CCI dependent mediation pathway between COVID-19 hospital caseload and TB CFR (COVID-19 hospital caseload → CCI → in-hospital TB CFR). The effect of mediation was estimated by multiplying the weights of edges in each mediation pathway. Thus, M1 = α_1_ * b_1,_ M2 = α_1_ * d_21_ * b_2_, and M3 = α_2_ * b_2_ [24]_._

In the model, both COVID-19 and TB caseload were log-transformed using base 2 due to their exponential nature of growth and to simplify interpretation. With log base 2, for every unit increase in the transformed COVID-19 or TB caseload corresponds to a doubling of its original value. For interpretation, the coefficients of these caseloads are transformed to a power of 2, which indicates how many percentage of the outcome variables would change by doubling the caseload. The CCI score was transformed by multiplying by 10 in the model to account for its small numeric range. For interpretation, this allows us to estimate the percentage change in TB CFR associated with every 0.1 score increase in the CCI of TB patients. This approach enhances the model’s sensitivity and can capture subtle shifts in CCI score that may be clinically significant, particularly in large-scale studies.

Bootstrap resampling of the original data was used to test the rigorous significance of all mediation pathways simultaneously. We performed 5,000 bootstrap resamples of the dataset, using the same resampling method for each iteration to calculate all regression and mediation effects. This approach was used to estimate 95% confidence intervals for the mediation effects. If both the upper and lower bounds of a confidence interval shared the same sign, the mediation or regression effect was considered significant (i.e., different from 0). Adjusted R^2^ was used to measure the fitness of the models. The “Lavaan” package in R software was used for serial mediation analysis [25]. R software version 4.1.3 [21] was used to create the ITS analysis figures and Microsoft PowerPoint was used to create the serial mediation analysis figure.

## Results

In Table 1, there was a total of 168,332 new TB patients admitted to the hospital during 2017–2022 (99,936 before COVID-19 pandemic and 68,396 during COVID-19 pandemic). It shows number of males was about 2.3 times higher than that of females. Adult and elderly age groups each contributed almost half of the patients, whereas the children age group was minimal. Pulmonary TB, both bacteriologically confirmed and clinically diagnosed, accounted for the majority of TB diagnoses. There are significant differences in the background characteristics of newly hospitalised TB patients before and during the COVID-19 pandemic. However, these statistical significances were due to large sample size, while the absolute degrees of the differences were relatively small. The disease severity (CCI score) and mortality were increased during the pandemic.

**Table 1:** Background characteristics, severity and mortality of TB patients hospitalised before and during COVID-19 period

Patients’ clinical profile	Number of new hospitalised TB patients			
	Overall period	Before and during COVID-19 period		
	Jan 2017- Sep 2022 (*N* = 168,332)	Before COVID-19 (Jan 2017- Feb 2020) (*N* = 99,936)	During COVID-19 (Mar 2020- Sep 2022) (*N* = 68,396)	*p*-value
**Gender**				< 0.001
Female	49,772 (29.6)	29,952 (30.0)	19,820 (29.0)	
Male	118,560 (70.4)	69,984 (70.0)	48,576 (71.0)	
**Age group (years)**				< 0.001
< 15	2406 (1.4)	1464 (1.5)	942 (1.4)	
15—59	85,522 (50.8)	51,242 (51.3)	34,280 (50.1)	
≥ 60	80,404 (47.8)	47,230 (47.3)	33,174 (48.5)	
**ICD-10 TB diagnosis**				< 0.001
Pulmonary TB (Bacteriologically confirmed)	72,359 (43.0)	43,369 (43.4)	28,990 (42.4)	
Pulmonary TB (Clinically diagnosed)	69,618 (41.4)	41,199 (41.2)	28,419 (41.6)	
TB of nervous system	5481 (3.3)	3203 (3.2)	2278 (3.3)	
TB of other organs	14,435 (8.6)	8731 (8.7)	5704 (8.3)	
Miliary TB	6439 (3.8)	3434 (3.4)	3005 (4.4)	
Charlson Comorbidity Index (CCI)^*^				< 0.001
Mean (SD)	0.64 (1.2)	0.61 (1.2)	0.67 (1.2)	
0	110,438 (65.6)	66,798 (66.8)	43,640 (63.8)	
1	35,310 (21.0)	20,258 (20.3)	15,052 (22.0)	
2	11,139 (6.6)	6333 (6.3)	4806 (7.0)	
≥ 3 to 29	11,445 (6.8)	6547 (6.6)	4898 (7.2)	
**Mortality**	11,501 (6.8)	6145 (6.1)	5356 (7.8)	< 0.001

^*^Charlson comorbidity index (CCI) score ranged from 0–29. The higher score predicts increased mortality by weighing different comorbid conditions to measure the severity of patient[19]; TB, Tuberculosis

### ITS analysis of TB hospital caseload during COVID-19 pandemic in Thailand

**Fig. 2:**
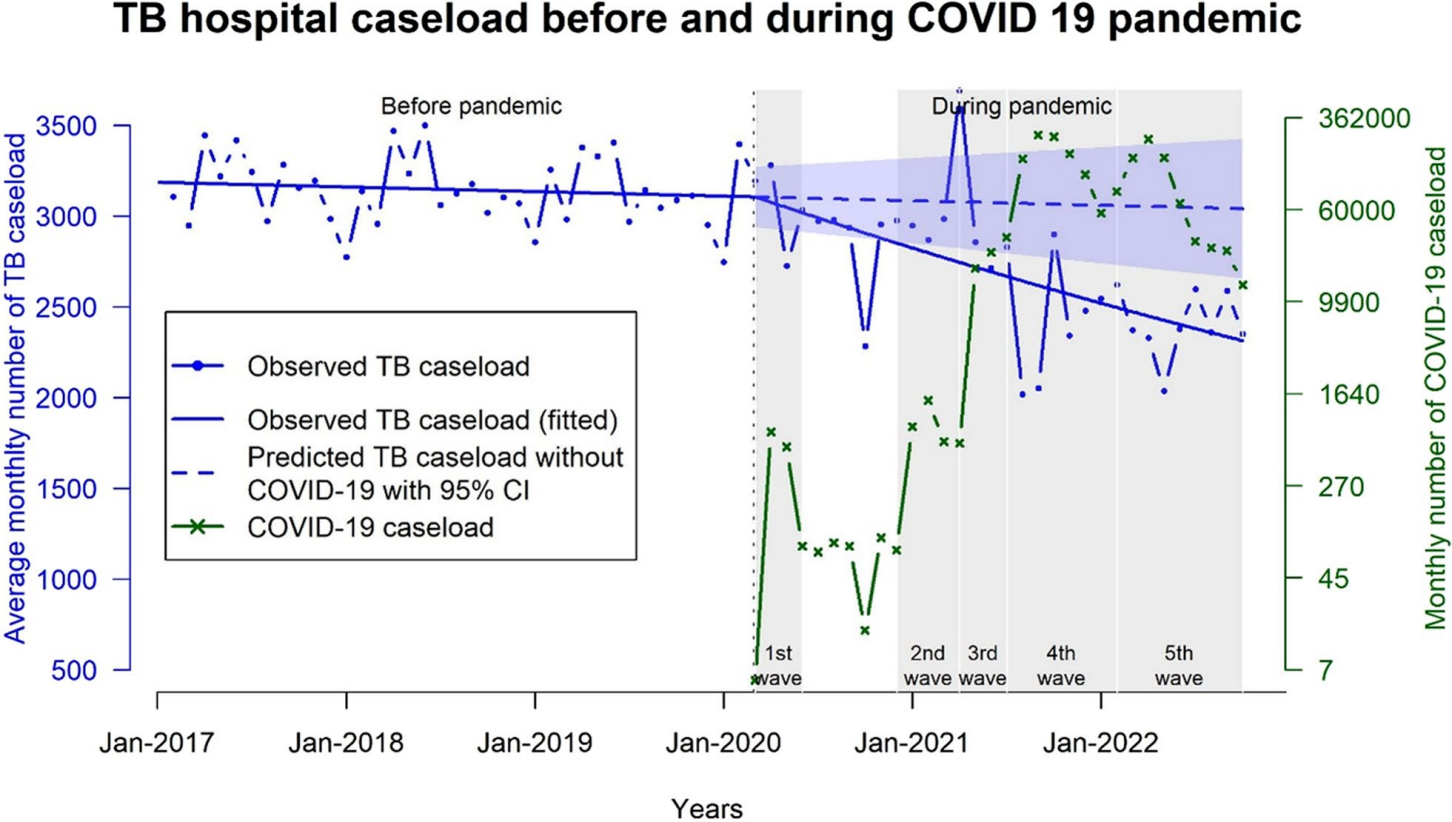
Trends of average monthly hospital caseload of TB and COVID-19 between 1st and 5th outbreaks of COVID-19. TB, Tuberculosis

Figure 2 shows that the trend of TB caseload before COVID-19 pandemic was quite stable, although there was some seasonal variation. During COVID-19 pandemic, the observed TB caseload trend continuously decreased at an average of 1% per month (*p*-value = < 0.001). (Supplementary Table 1). The decreasing trend of TB caseload was seen in each health region after stratification (Supplementary Table 2). In contrast, the COVID-19 caseload trend steeply increased from the 1st to the 4th waves and remained steady for a few months before decreasing in the latter period of the 5th wave (Fig. 2).

In Table 2, there was a 12.83% decrease in the average monthly TB caseload between observed and counterfactual during the pandemic period. The largest reductions were found during the fourth and the fifth outbreaks.

**Table 2:** Changes in average monthly TB hospital caseload between 1st and 5th outbreaks of COVID-19

Average monthly hospital TB caseload	Outbreak period					
	Overall period	1st	2nd	3rd	4th	5th
	Mean (95% CI)					
Observed (actual)	2677.2 (2537.8, 2816.6)	3012.7 (2320.6, 3704.8)	3123.5 (2516.4, 3730.6)	2800.0 (2609.9, 2990.2)	2422.1 (2131.3, 2712.9)	2376.0 (2230.7, 2521.3)
Counterfactual	3071.2 (2793.7, 3348.8)	3100.2 (2920.6, 3279.9)	3080.5 (2835.3, 3325.7)	3073.3 (2803.3, 3343.3)	3062.9 (2757.3, 3368.5)	3047.5 (2688.3, 3406.7)
Percent difference^*^	**−12.83** **(−9.16,** **−15.89)**	−2.83 (−20.55, 12.95)	1.40 (−11.25, 12.17)	**−8.89** **(−6.90,** **−10.56)**	**−20.92** **(−19.46,** **−22.70)**	**−22.03** **(−17.02,** **−25.99)**

^*^Percent difference, ((Observed – Counterfactual)/Counterfactual) × 100; TB, Tuberculosis; CI, Confidence interval; The bold values, Statistically significant

### ITS analysis of TB hospital case fatality rate (CFR) during COVID-19 pandemic in Thailand

**Fig. 3:**
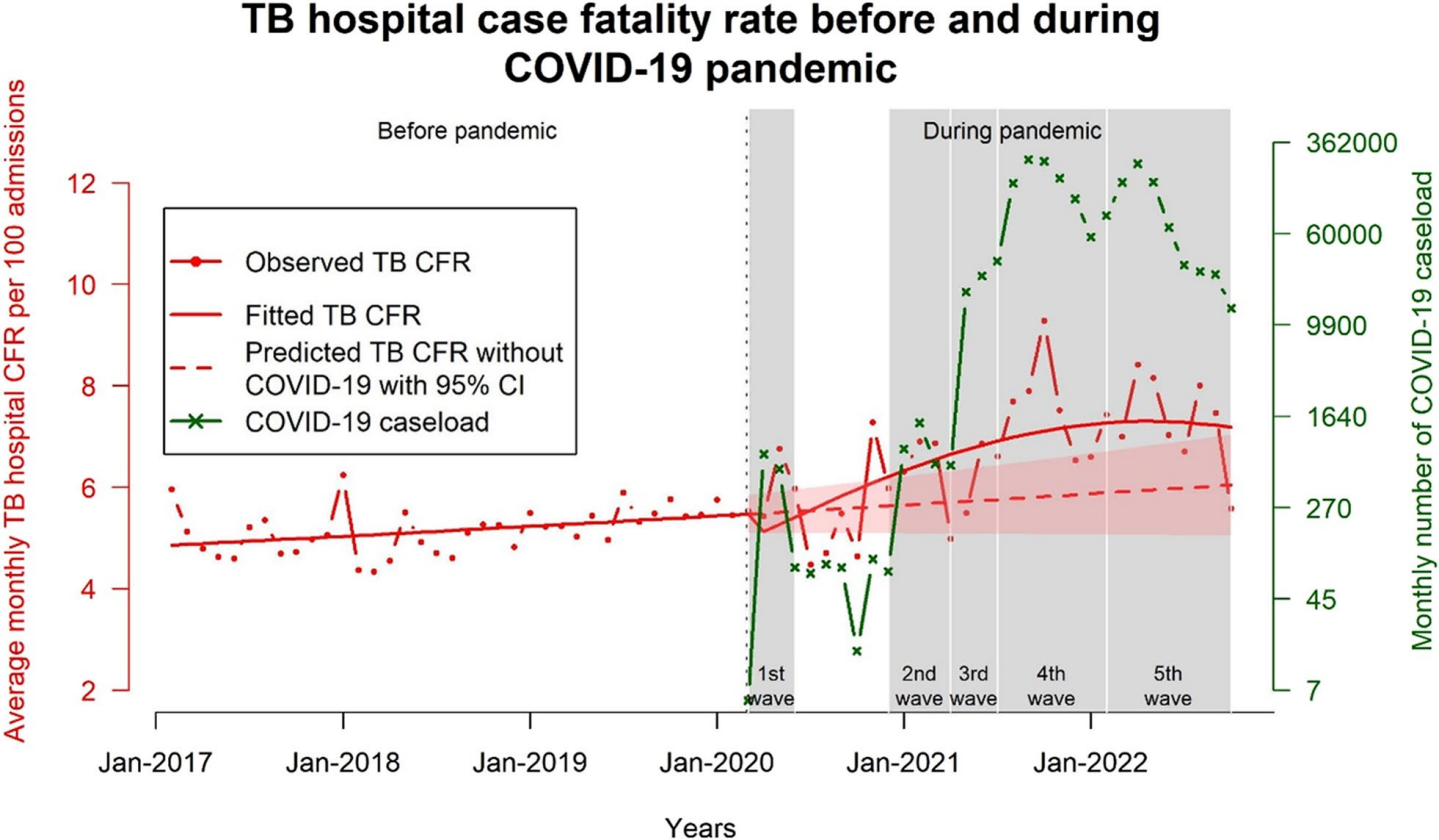
Trend of average monthly TB hospital CFR and COVID-19 caseload between 1st and 5th outbreaks of COVID-19. TB, Tuberculosis, CFR, case fatality rate

Figure 3 shows that the TB hospital CFR slightly increased over time before the COVID-19 pandemic. It shows an increasing trend (*p*-value = 0.004) between the first and fourth outbreaks, followed by a downward curvilinear trend (*p*-value = 0.03) (Supplementary Table 1) during the fifth wave. Moreover, a similar trend between TB CFR and average COVID-19 caseload was detected during COVID-19 pandemic, suggesting a possible relationship. After stratification, a consistent pattern was seen in most regions (Supplementary Table 2), which was well aligned with the overall findings at the national level.

The average monthly TB hospital CFR during the pandemic is summarized in Table 3. Overall, a significant increase in TB CFR (14.08%) during COVID-19 pandemic compared to the counterfactual, highest in the fourth (30.19%) and slightly downward in the fifth outbreaks (21.7%).

**Table 3:** Changes in average monthly TB hospital CFR between 1st and 5th outbreaks of COVID-19

Average monthly hospital TB CFR/100 admissions	Outbreak period					
	Overall period	1st	2nd	3rd	4th	5th
	Mean (95% CI)					
Observed (actual)	6.56 (6.17, 6.96)	6.01 (5.76, 6.26)	6.19 (5.68, 6.70)	6.31 (4.80, 7.82)	7.59 (6.07, 9.11)	7.27 (6.53, 8.01)
Counterfactual	5.75 (5.08, 6.43)	5.51 (5.09, 5.92)	5.68 (5.09, 6.26)	5.74 (5.08, 6.40)	5.83 (5.07, 6.59)	5.97 (5.05, 6.89)
Percent difference^*^	**14.08** **(8.32, 21.46)**	**9.08** **(5.74,** **13.16)**	**8.97** **(7.03, 11.59)**	9.93 (−5.51, 22.19)	**30.19** **(19.72, 38.24)**	**21.78** **(16.26, 29.31)**

^*^Percent difference, ((Observed – Counterfactual)/Counterfactual) × 100; TB, Tuberculosis; CFR, Case fatality rate; CI, Confidence interval; The bold values, Statistically significant

### Association between COVID-19 caseload with TB hospital caseload and in-hospital TB CFR between March 2020 and September 2022

The study observed when COVID-19 caseload increased, the TB caseload decreased simultaneously in the same month (r = − 0.60, *p*-value = < 0.001). Supplementary Fig. 5 indicates no lag effect. Conversely, with higher COVID-19 caseload, a higher TB hospital CFR was observed (r = 0.74, *p*-value = < 0.001). Supplementary Fig. 7 shows the positive correlation with no lag effect.

In stratified analysis across 13 health regions, the majority of correlation coefficients between COVID-19 caseload and both TB hospital caseload and TB CFR peaked during the same month (Supplementary Fig. 6 and 8), which contributes to the overall national level association (Supplementary Fig. 5 and 7). The + 1 or − 1-month lag in a few regions may be due to slight differences in the timing of pandemic impact across regions.

### Serial mediation effect of TB caseload and severity of TB patient between COVID-19 hospital caseload and in-hospital TB CFR

**Fig. 4:**
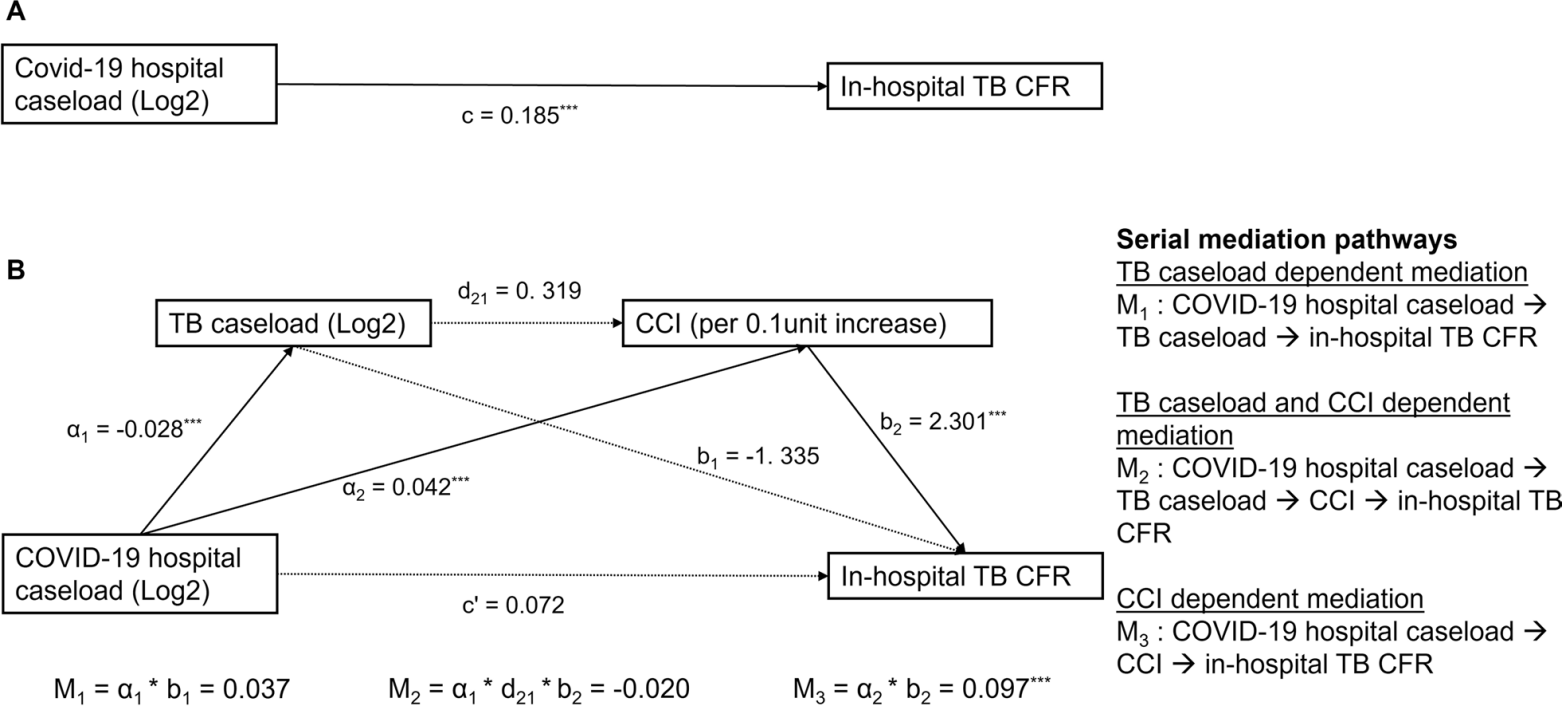
Serial mediation model. Note. Diagram of model pathways showing total effect of COVID-19 hospital caseload on in-hospital TB CFR (Fig. 4 A) and serial mediation model including three mediation pathways (Fig. 4B). The strength of a mediation pathway (i.e., M_1_, M_2_, M_3_) is the multiplicative product of the components in the pathway, as indicated at the bottom of Fig. 4B. ^*^ p-value < 0.5; ^**^ p-value < 0.01; ^***^ p-value < 0.001; TB, Tuberculosis; CCI, Charlson comorbidity index; CFR, Case fatality rate

Figure 4A shows the total effect of COVID-19 hospital caseload on in-hospital TB CFR, and Fig. 4B presents the serial and individual mediation effects of TB caseload and CCI between COVID-19 hospital caseload and in-hospital TB CFR. In Fig. 4A, the total effect shows a two times increase in the COVID-19 caseload significantly increased TB deaths by 0.19 per 100 TB admissions. In Fig. 4B, COVID-19 hospital caseload has a significant negative effect (α1) on TB caseload but a positive effect (α2) on the CCI score. And the CCI score has a significant positive effect (b2) on TB CFR. When the COVID-19 caseload doubled, TB caseload changes by 2^−0.028^ = 0.98% or decreased by 2%, while the CCI score of TB patients increased by 2^0.04^ = 1.03 or 3%. And, for every 0.1 score increase in CCI, the CFR of TB will be increased by 2.3 deaths per 100 TB admissions. However, no significant effect of TB caseload on CCI score and TB CFR was detected.

Figure 4B also shows three mediation pathways. M_1_ indicates the TB caseload dependent mediation pathway between COVID-19 hospital caseload and TB CFR. M_2_ shows both TB caseload and CCI dependent serial mediation pathway between COVID-19 hospital caseload and TB CFR. And M_3_ expresses the CCI dependent mediation pathway between COVID-19 hospital caseload and TB CFR. Among these three mediation models, a significant mediation effect was found with the CCI score only following M_3_ pathway, and no significant mediation effect of TB caseload was detected. Additionally, there is no significant direct effect of COVID-19 hospital caseload on TB CFR after controlling for the mediating effect of both TB caseload and CCI score. These findings suggest there was no direct association between COVID-19 caseload and TB CFR, but it was mediated by CCI score.

In model evaluation, the adjusted *R*^2^ value of the total effect was 0.53, while that of the model with mediators was 0.70, which reflects that the mediation model explained more variability of TB CFR than the model without mediations.

## Discussion

During COVID-19 pandemic, there was a significant decrease in the monthly TB hospital caseload, but an increase in the in-hospital TB CFR. COVID-19 hospital caseload was negatively correlated with the former but positively correlated with the latter without a lag interval. We demonstrated that COVID-19 hospital caseload was not directly associated with higher hospital TB CFR but mediated by the level of severity of TB cases.

Overall, there was a 12.8% decrease in the TB hospital caseload between 2020 and 2022 due to COVID-19 pandemic. A significant reduction was seen during the third, fourth and fifth outbreaks. This finding is in line with the previous study done in Zambia and China reporting significant declines in the number of TB case-notifications and TB incidence during COVID-19 pandemic [4, 26]. The studies done in non-COVID-19 hospitalised patients in Italy and US also reported decreases in hospital caseloads during the pandemic [27, 28]. One reason might be the deferment of patients with less severe health problems from admission to the hospital due to the relocation of healthcare resources for the increasing COVID-19 caseload [29]. A second reason might be the patients’ avoidance of seeking medical care in response to the fear of being infected with COVID-19 during the surge of the outbreak [30, 31]. These hypotheses were supported by our finding on a significant negative correlation (r =- 0.60) between COVID-19 hospital caseload and TB hospital caseload without lag effect. The major reductions in TB hospital caseload of 21.7% and 22.9% in our study were found during the fourth and fifth outbreaks, respectively, coinciding with the significant peaks in COVID-19 caseload.

It can be argued that the decrease in number of TB patients presented to hospitals might be due to travel restrictions and limited transportation services during the lockdown period of the pandemic. However, the impact of lockdown may be less pronounced in Thailand because the nationwide lockdown was implemented during the first outbreak, and only partial lockdowns were imposed during the subsequent outbreaks that allowed visits to healthcare facilities [17].

While TB hospital caseload decreased, a significant 14.9% increase in TB hospital CFR was observed during COVID-19 pandemic. This is in line with the previous study in China, which stated that the TB mortality was increased over the course of the pandemic, although TB incidence was decreased [4]. This effect was also seen in WHO European region where nearly 7000 excess deaths of TB patients occurred during the pandemic [32]. It was mentioned that disruption of the healthcare system with decreased performance of diagnosis and treatment efforts, led to the increase in mortality of TB patients [32]. Near the end of the study period, both COVID-19 caseload and TB CFR decreased. The former decline was due to the epidemiological nature of the pandemic while the latter could be explained by the relationship between the COVID-19 caseload and selection bias of TB severity in the model.

In our study, a significant strong positive correlation was seen between COVID-19 hospital caseload and TB hospital CFR (r = 0.75). The percent increase of TB hospital CFR during sub-periods of the pandemic was proportionate to the COVID-19 caseload. The mortality took place soon within the same month when having a high COVID-19 caseload without a significant lag. It has been assumed that increase in CFR of TB patients admitted to hospital might not only be associated with the poor quality of care due to shifting of healthcare resources to COVID-19 departments, but also be due to patients with relatively less severe condition being reluctant to go to hospital due to fear of contracting COVID-19, leaving only severe cases, which led to a higher hospital case fatality rate [33–36].

Regarding the severity of TB patients admitted to the hospital during COVID-19, the number of TB patients with a CCI score ≥ 1 increased from 33.2% before COVID-19 to 36.2% during COVID-19. This finding was in line with the critical care resources allocation guideline in Thailand developed by the International Health Policy Program, in which CCI is one of the major criteria to prioritise critical healthcare resources to mitigate the depletion of resources during the height of the COVID-19 pandemic [37]. Studies done in Belgium and Korea reported that the severity of non-COVID-19 patients presenting to emergency department and intensive care unit increased significantly during the COVID-19 pandemic [35, 38].

In our study, the direct relationship between COVID-19 caseload and TB hospital CFR disappeared after TB caseload and severity of TB were added to the mediation model. This indicates that the initial relationship was confounded by TB case severity differentials across the COVID-19 period. In the peak of the pandemic, less severe TB cases might choose to stay or be treated at home. Severe cases eventually need hospitalisation while they are more likely to die. This selection differential declined as the COVID-19 caseload declined. Previous studies done in China, Spain and the US also reported that hospitalised non-COVID-19 patients during the pandemic had a more severe status than those admitted before the pandemic [39–41]. This is also supported by our descriptive findings on the shift in severity of case mix contributing to admissions during the pandemic. Had the two additional variables been ignored, one would misinterpret that the COVID-19 caseload, which is the proxy of health system quality of care, was the main cause of TB CFR. Our findings reflect that the increased mortality of TB patients during the pandemic was explained by the admission of severe cases with a subsequent higher risk of mortality, but not by the quality of care in hospitals.

The lesser effect of COVID-19 pandemic on the quality of care in hospitals might be associated with the proactive measures, such as improvement of resilience capacity and expansion of field hospitals to decrease workload following the pandemic. A study exploring the impact of COVID-19 on non-communicable diseases in Thailand endorsed the assumption that the medical supply for healthcare facilities was less affected by COVID-19 pandemic due to the resilience capacity of the healthcare facilities [42]. In addition, to reduce the workload of COVID-19 in high-level hospitals during the surge of outbreaks, the provincial-level hospitals initially managed the identified COVID-19 cases and repatriated them to community isolation units and field hospitals expanded in their hometown. Thus, healthcare staff from the District Public Health, Subdistrict Health Promoting Hospital, Village Health Volunteers, and community leaders shared the workload of COVID-19 cases [43]. These imply that enhancing the healthcare system’s resilience and decentralising care are likely remedies to retain the quality of care in hospitals during the pandemic.

Countries with different COVID-19 trajectories including not only high TB burden countries in Asia and Africa but also low TB burden countries in the European region reported increase in TB mortality during the pandemic [32, 44]. For example, in Italy, despite not being a high TB burden country, TB diagnosis and care services were severely jeopardised as resources were redirected to COVID-19 response, leading to a rise in TB mortality during the initial period when COVID-19 hit worst compared to other European countries [45]. It highlights the importance of ensuring uninterrupted TB care during pandemics, regardless of a country’s TB burden.

Our study suggests that during pandemics, healthcare systems must be prepared for a likely increase in the admission of severe TB cases. This highlights the importance of ensuring access to TB care, amid public health emergencies. While decentralised COVID-19 care and sharing workload across health facilities maintain service for non-COVID-19 diseases, other innovative strategies such as promoting telehealth services, reinforcing human workforce by increasing trained personnel, and prioritising services for emergency and basic clinical services proved effective alternatives for resource allocations [46–48]. These resource allocation strategies should be proactively implemented to sustain health services in future pandemics. Additionally, health systems should emphasise early detection and effective treatment of all TB cases to reduce severe TB cases before the pandemic arrives.

### Strengths and limitations

There are some limitations in the study. Although the THIP database well covers all COVID-19 admissions and TB admissions, it still might miss some patients who died of TB soon after being discharged from the hospital. This database may not represent TB cases who were not able to access hospitalised care during the pandemic. COVID-19 pandemic did not equally affect all areas in Thailand and the quality of care in all areas could be varied. Nevertheless, the study addressed the policy-relevant question on the nationwide impact of COVID-19 on the outcome of TB patients in hospitals. In our study, we did not specifically account for the TB-COVID-19 co-infection attribution to increased mortality. Notably, we found that the direct effect of COVID-19 caseload on TB CFR vanished after adjusting for TB caseload and severity of TB patients. All these suggest that TB caseload and severity of TB played more important roles than co-infection on TB CFR. The mortality rate of TB patients only accounts for deaths in hospitals and may underestimate the total mortality because of the decreased rate of admission during the COVID-19 period. The mortality of TB patients who were not admitted to hospitals needs to be explored to see the impact of COVID-19 on TB patients who did not receive hospital care. There are also strengths in the study. The study utilised the THIP database which is a high-quality hospital admission dataset without significant missing information, as it undergoes validation for reimbursement purposes. The study explored a series of changes in hospital caseload and CFR during all major periods of COVID-19, including nationwide lockdown and subsequent outbreaks.

## Conclusion

In Thailand, the COVID-19 pandemic decreased the hospital caseload of TB patients. The observed increase in the in-hospital TB CFR was associated with increased admission of cases comorbid with higher risk of mortality, and not due to worsening of the quality of care.

## Supplementary Information

Additional file 1.

## Data Availability

The data that support the findings of this study are available from the Thai Health Information Portal (THIP), but restrictions apply to the availability of these data, which were used under license for the current study and are not publicly available. Data are, however, available from the authors upon reasonable request and with permission of THIP.

## Abbreviations

TB: Tuberculosis
COVID-19: Coronavirus disease 2019
CFR: Case fatality rate
THIP: Thai Health Information Portal
NHSO: National Health Security Office
NSTDA: National Science and Technology Development Agency
NBT: National Biobank of Thailand
ICD-10: International Classification of Diseases, tenth revision
CCI: Charlson comorbidity index
ITS: Interrupted time series
RR: Rate ratio
CI: Confidence interval
WHO: World Health Organization
