# Supplementary material for: Impact of COVID-19 pandemic, and the mediating role of hospital caseload and severity on mortality of hospitalised tuberculosis patients in Thailand
Source: Glob Health Res Policy. 2025 Aug 25;10:37. doi: 10.1186/s41256-025-00437-7 (PMC12376735; doi:10.1186/s41256-025-00437-7)
Supplement: Supplementary file 1 — Additional file 1. [file 41256_2025_437_MOESM1_ESM.docx]

**Supplementary materials**


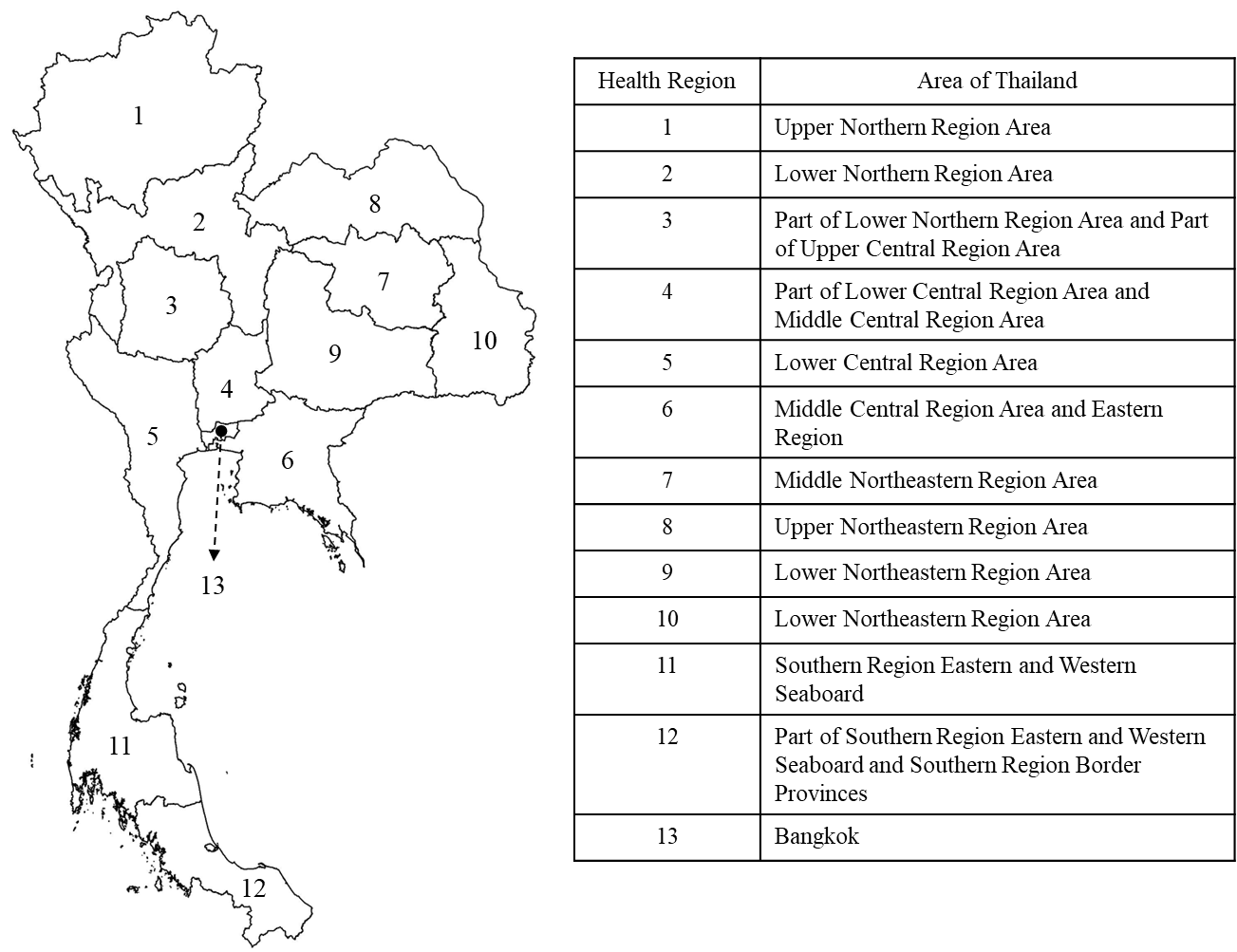


**Supplementary Fig. 1 Map of 13 health regions in Thailand.**

**
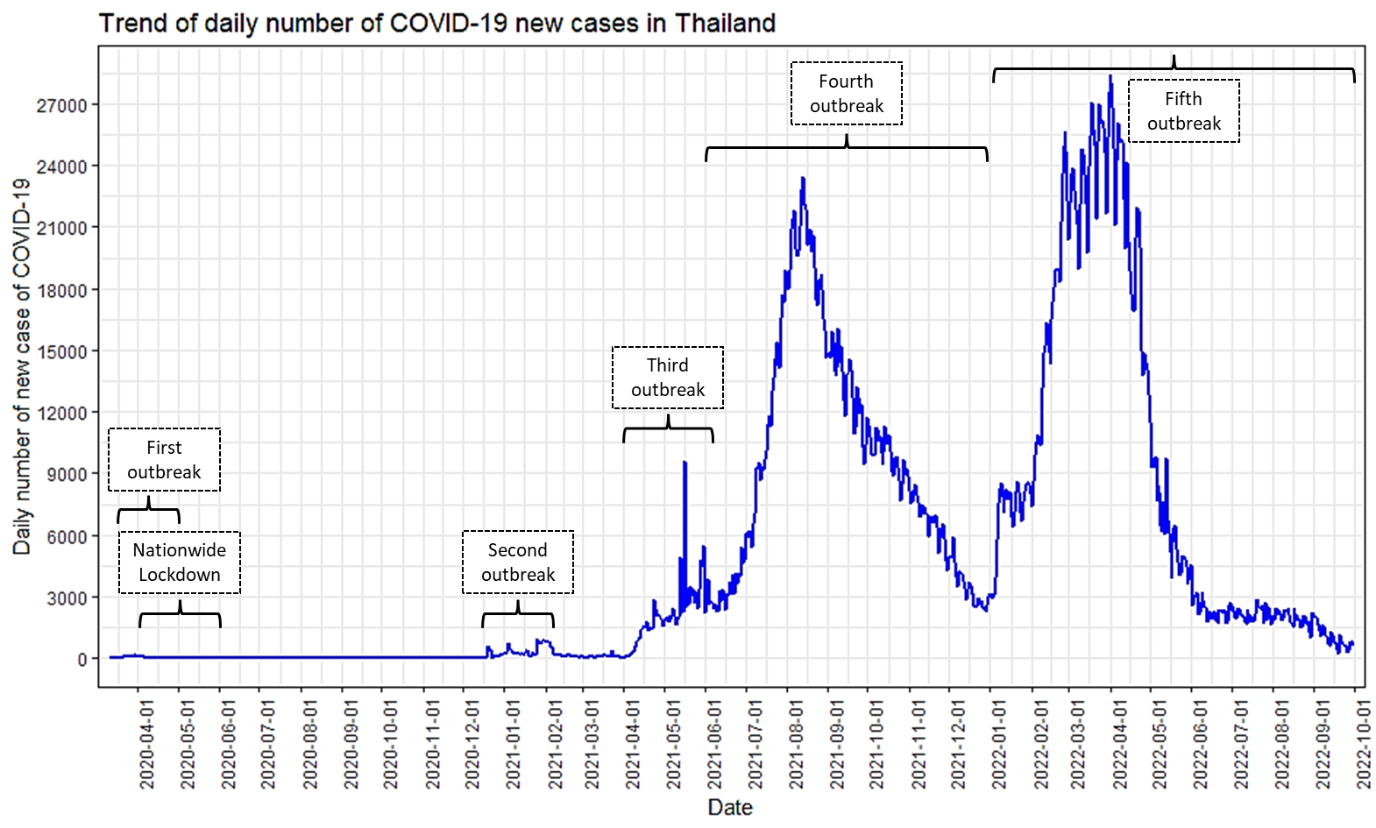
**

**Supplementary Fig. 2 Daily number of new cases of COVID-19 during nationwide lockdown and outbreaks.** The Royal Thai Government Gazette published an announcement on COVID-19 as state of emergency in March 2020 followed by first outbreak. The declaration for national lockdown was started in April until June 2020 after the end of first outbreak. Generally, the timeline of COVID-19 pandemic period in Thailand can be separated into five phases: (1) the nationwide lockdown in the first outbreak period and (2) four outbreak periods with partial lockdown.

| **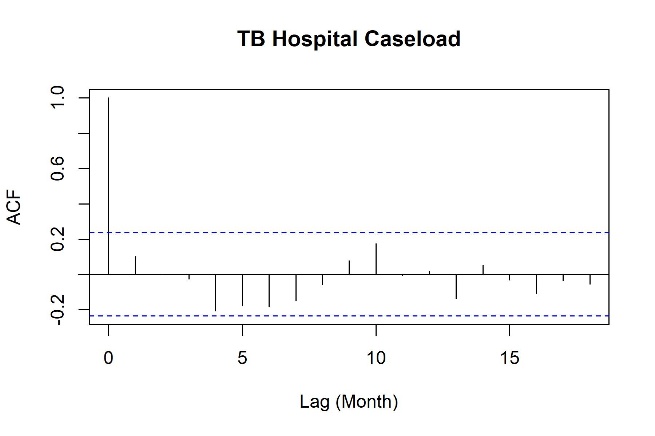** | **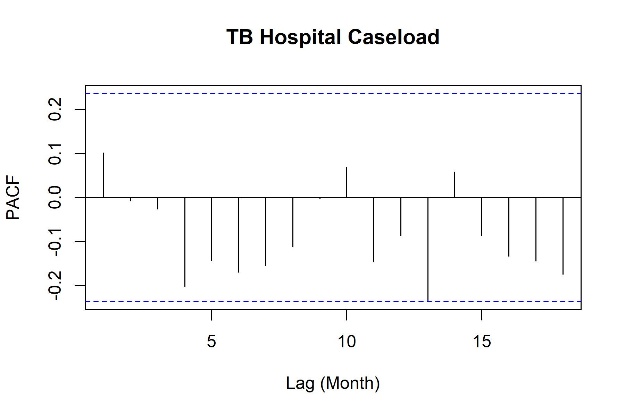** |
| --- | --- |

**Supplementary Fig. 3 ACF and PACF plot for interrupted time series model of TB hospital caseload**

| **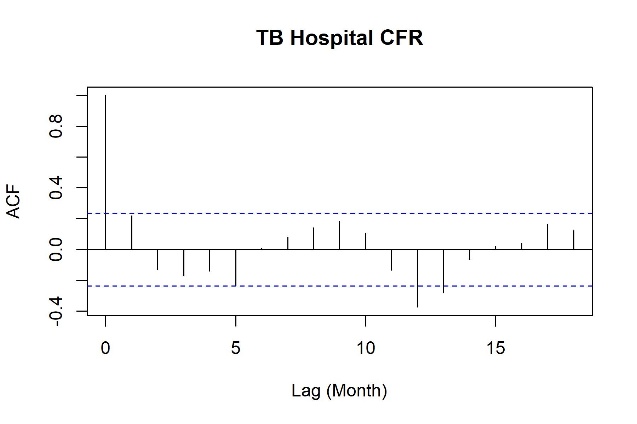** | **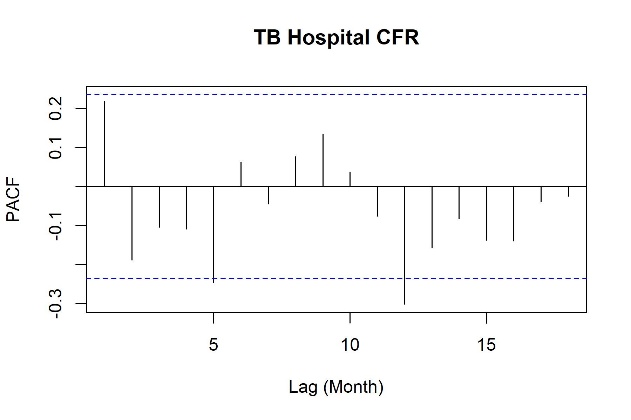** |
| --- | --- |

**Supplementary Fig. 4 ACF and PACF plot for interrupted time series model of TB hospital case fatality rate (CFR)**


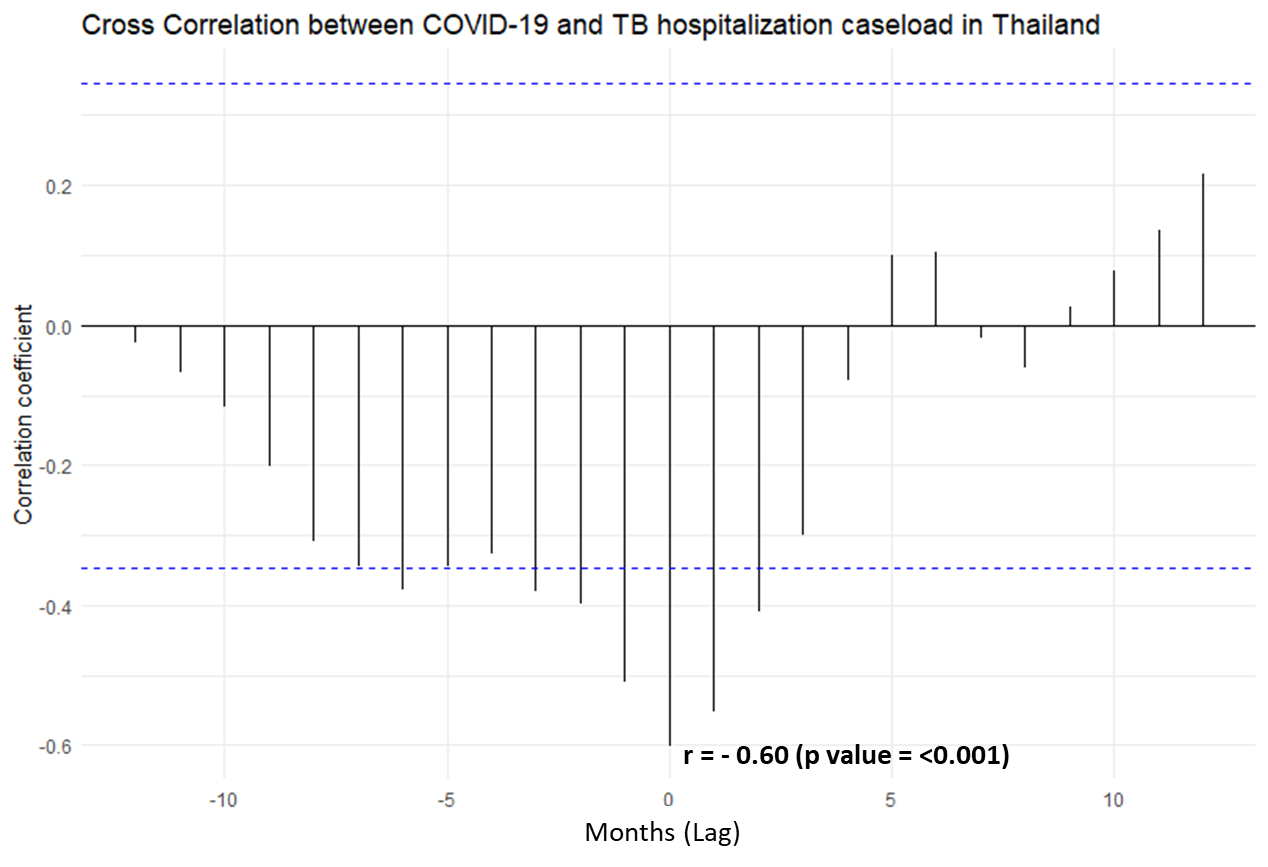


**Supplementary Fig. 5 Correlation between COVID-19 caseload and TB hospital caseload including lag time in Thailand**


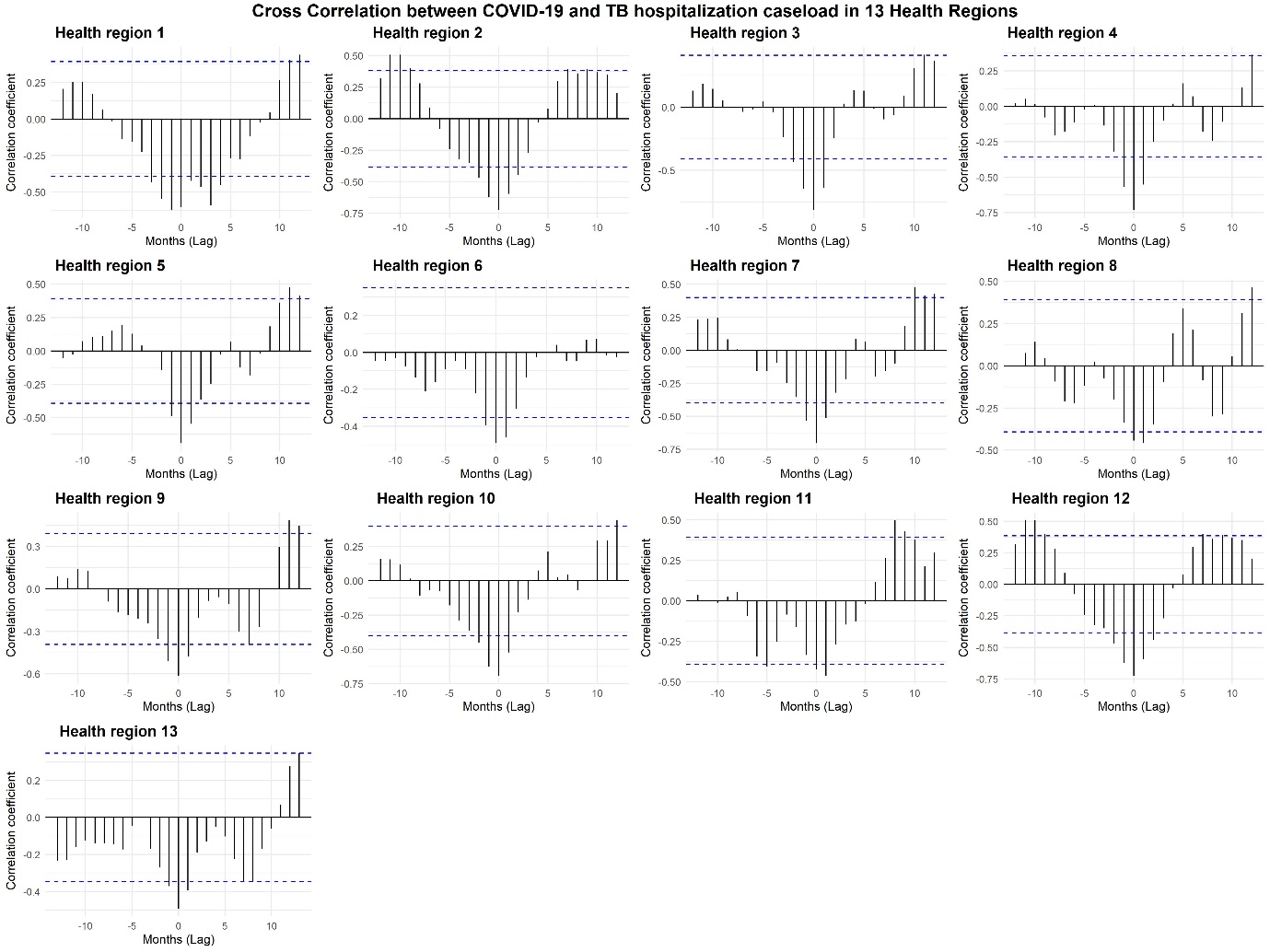


**Supplementary Fig. 6 Correlation between COVID-19 and TB hospital caseload including lag time in 13 Health Regions**


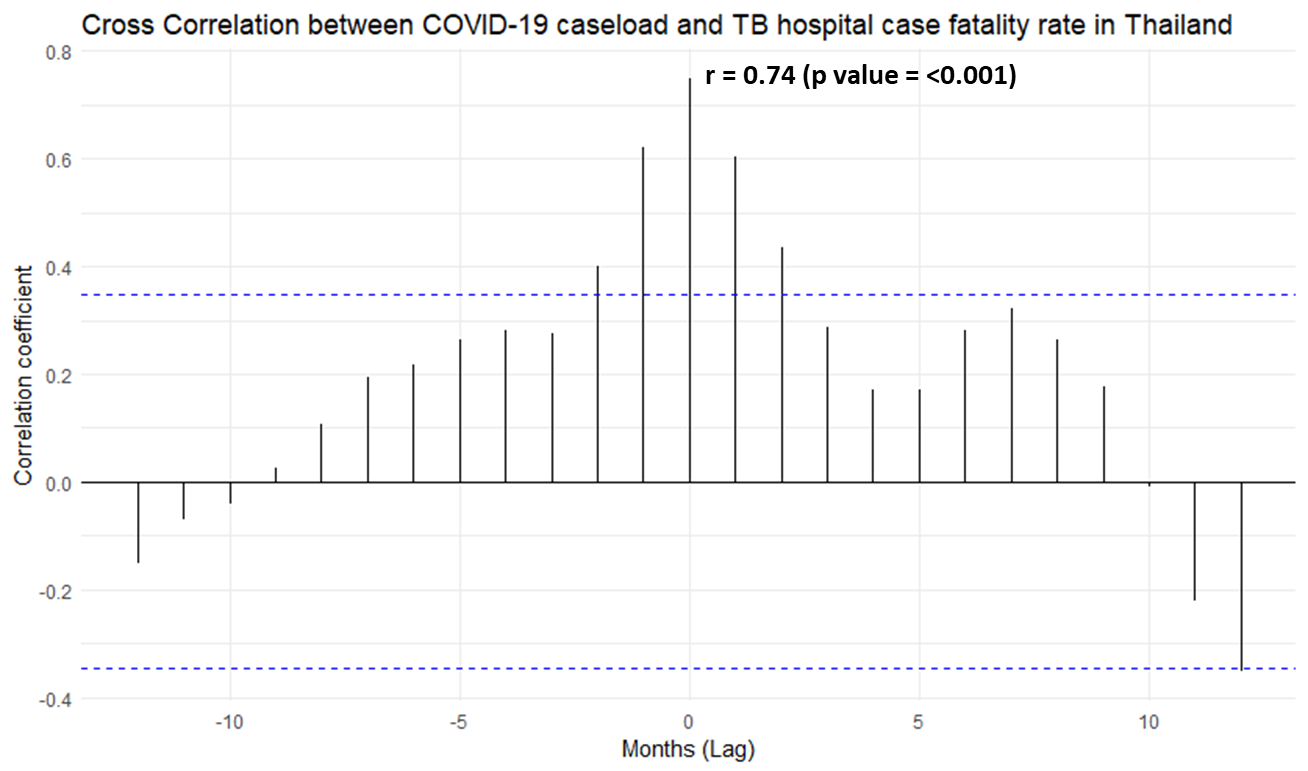


**Supplementary Fig. 7 Correlation between COVID-19 caseload and TB hospital case fatality rate including lag time in Thailand**


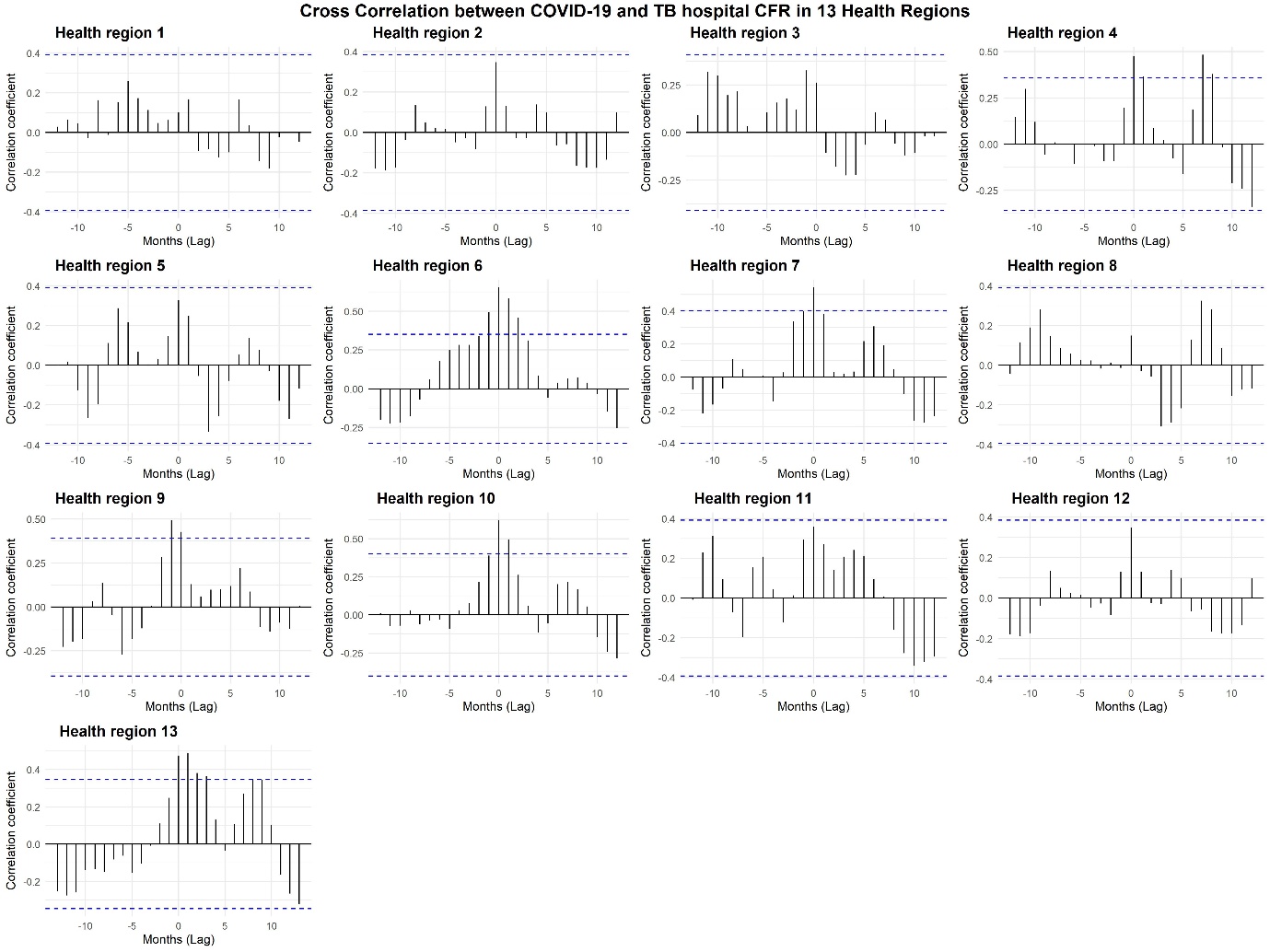


**Supplementary Fig. 8 Correlation between COVID-19 caseload and TB hospital case fatality rate including lag time in 13 Health Regions**

**Supplementary Table 1. Changes in trend of monthly TB caseload and in-hospital TB case fatality rate during COVID-19**

|  | **TB caseload** | **TB case fatality rate** |
| --- | --- | --- |
| **Model output** |  |  |
| Immediate change,  RR (95% CI) | 0.977 (0.900, 1.061) | 0.509 (0.196, 1.320) |
| Change in trend during pandemic, RR (95% CI) | **0.991 (0.987, 0.996)** ^*^ | **1.027 (1.008, 1.046)** ^*^ **0.999 (0.999, 1.000)**^#^ ^*^ |

Abbreviation: RR – Rate ratio;

# - Quadratic term, The bold values – Statistically significant, ^*^ p-value < 0.05, ^*^ p-value < 0.01

**Supplementary Table 2. Changes in trend of monthly TB caseload and in-hospital TB case fatality rate in 13 health regions during COVID-19**

|  | **COVID-19 pandemic** | |
| --- | --- | --- |
|  | **Immediate change**  **RR (95% CI)** | **Change in trend during pandemic**  **RR (95% CI)** |
| **TB Hospital caseload** |  |  |
| Health Region 1 | 0.993 (0.902, 1.094) | **0.985 (0.980, 0.990)^***^** |
| Health Region 2 | 1.092 (0.954, 1.249) | **0.986 (0.979, 0.993)^***^** |
| Health Region 3 | 0.921 (0.820, 1.035) | **0.987 (0.981, 0.993)^***^** |
| Health Region 4 | 1.006 (0.890, 1.137) | **0.990 (0.984, 0.997)^**^** |
| Health Region 5 | 0.952 (0.856, 1.059) | 0.997 (0.991, 1.002) |
| Health Region 6 | 0.942 (0.844, 1.051) | **0.991 (0.986, 0.997)^**^** |
| Health Region 7 | 1.061 (0.960, 1.173) | 0.999 (0.994, 1.004) |
| Health Region 8 | 0.977 (0.883, 1.082) | 0.997 (0.992, 1.002) |
| Health Region 9 | 0.990 (0.902, 1.087) | **0.989 (0.985, 0.994)^***^** |
| Health Region 10 | 1.057 (0.939, 1.190) | **0.989 (0.983, 0.995)^***^** |
| Health Region 11 | 1.025 (0.899, 1.169) | **0.989 (0.982, 0.995)^**^** |
| Health Region 12 | 0.913 (0.791, 1.053) | **0.992 (0.985, 0.999)^*^** |
| Health Region 13 | 1.042 (0.918, 1.183) | **0.986 (0.980, 0.993)^***^** |
|  |  |  |
| **TB Hospital CFR** |  |  |
| Health Region 1 | 1.095 (0.845, 1.420) | 0.999 (0.985, 1.012) |
| Health Region 2^#^ | 0.919 (0.625, 1.351) | 1.049 (1.000, 1.101) |
| Health Region 3 | 1.014 (0.739, 1.390) | **1.026** (**1.010**, **1.043**)**^**^** |
| Health Region 4 | 1.097 (0.875, 1.375) | 1.008 (0.996, 1.020) |
| Health Region 5 | 1.030 (0.809, 1.311) | 1.005 (0.993, 1.018) |
| Health Region 6^#^ | 0.751 (0.560, 1.007) | **1.046** (**1.008**, **1.086**)**^*^** |
| Health Region 7^#^ | 0.751 (0.467, 1.206) | **1.070** (**1.011**, **1.132**)**^*^** |
| Health Region 8 | 1.285 (0.924, 1.786) | 1.015 (0.998, 1.032) |
| Health Region 9 | 1.009 (0.808, 1.260) | 1.004 (0.992, 1.016) |
| Health Region 10 | 1.055 (0.758, 1.468) | **1.019** (**1.002**, **1.036**)**^*^** |
| Health Region 11 | **0.674** (**0.468**, **0.970**)**^*^** | **1.021** (**1.002**, **1.040**)**^*^** |
| Health Region 12 | 0.978 (0.713, 1.343) | 0.998 (0.982, 1.015) |
| Health Region 13^#^ | **0.707** (**0.506**, **0.989**)**^*^** | **1.062** (**1.016**, **1.109**)**^**^** |

Abbreviation: RR – Rate ratio;

# - Quadratic term, The bold values – Statistically significant, ^*^ p-value < 0.05, ^*^ p-value < 0.01, ^***^ p-value < 0.001
